# Association between working time quality and self-perceived health: analysis of the 3rd Korean working conditions survey (2011)

**DOI:** 10.1186/s40557-017-0211-y

**Published:** 2017-11-13

**Authors:** Jaeyoup Jung, Gyuree Kim, Kyusung Kim, Domyung Paek, Sung-il Cho

**Affiliations:** Department of Occupational and Environmental Medicine, Graduate, School of Public Health, Seoul National University, Gwanak-ro 1, Gwanak-gu, Seoul, 151-600 Republic of Korea

**Keywords:** Self-perceived health, Working hours, Shift work, KWCS

## Abstract

**Background:**

Self-perceived health, a subjective assessment of one’s health condition, is an important health indicator at the level of quality of life. In this study, working time quality refer to job factors with qualitative aspects of working time. This study was conducted to investigate the association between working time quality and self-perceived health in paid workers in Korea.

**Methods:**

In this study, 35,902 paid workers were analyzed based on the 3rd Korean working conditions survey. For independent variables, working time quality (working at night, working in the evenings, working on Sundays, working on Saturdays, and working more than 10 h a day) were set as major job-related variables. Other occupational characteristics were divided into 6 groups and general characteristics were divided into 6 groups, and univariate analysis was conducted with self-perceived health, a dependent variable. Variables that had significance in the univariate analysis were used for multivariate logistic regression analysis.

**Results:**

In the univariate analysis using Chi-square test, variables showing significance in self-perceived health were age, income, education, occupation, employment type, work hours per week, and shift work. Working time quality showed a significant association with self-perceived health. After adjusting for these variables using logistic regression analysis, working at night, working in the evening, working on Sundays, and working more than 10 h a day showed significant association with self-perceived health.

**Conclusions:**

This study showed a statistically significant association between working time quality of employees with self-perceived health.

## Background

Health is a state of complete physical, mental and social well-being and not merely the absence of disease or infirmity [1]. Health is a resource for everyday life, not the objective of living. It is a positive concept emphasizing social and personal resources, as well as physical capacities [2].

Self-perceived health is a subjective assessment of one’s health which comprehensively reflects the individual’s overall health condition and sense of one’s health [3]. It is a health index that can be easily measured at a low cost [4, 5]. The reason why subjective assessment of one’s health is so important is because the level of an individual’s well-being not only affects the desire to live and quality of life but also has a strong association with morbidity and mortality [6].

If workers’ health is viewed from the perspective of physical aspects only, it will fail to capture the fundamental meaning of health set out above. Therefore, when assessing the health condition of workers affected by different job-related factors, the view needs to be extended to qualitative aspects, in addition to quantitative aspects. Self-perceived health is an often used index to represent general health status [7] that is known to predict future mortality [8, 9].

According to the current literature, the factors influencing self-perceived health are age, low socioeconomic status, education level, depression, social support, smoking, drinking, quality of sleep, and chronic disease [10]. Studies examining numerous variables related to self-perceived health have been conducted in Korea [11–14], and these variables have been studied from an occupational perspective.

Studies with an occupational perspective have examined the effects of different factors, including shift work [15], employment type [16], job satisfaction [17], and the number of work hours [18] on self-perceived health. What is different from the existing research is that we approached the qualitative aspect of time in the analysis of working time. Escaping from the normal pattern of work hours can be comprehended by the presence of shift work or the number of work hours as in previous studies. However, considering the concept of health in this study, different factors such as late work hours, working on weekends, and working long hours a day will affect the ‘health at the level of quality of life’ from the perspective of workers’ subjective standard of living. Therefore, the purpose of this study is to find out whether the qualitative factor of time in working hours is related to self-perceived health.

In this study, Working time quality refer to job factors with qualitative aspects of working time and the following characteristics were selected for this study: working at night, working in the evenings, working on Sundays, working on Saturdays, and working more than 10 h a day. This study was conducted to investigate the association between working time quality and self-perceived health in paid workers in Korea.

## Methods

### Study subjects

This study used data from the Korean Working Conditions Survey (KWCS), which was conducted in 2011 by the Occupational Safety and Health Research Institute (OSHRI) of the Korea Occupational Safety and Health Agency (KOSHA). The KWCS examined overall conditions of employment (form of labor, form of employment, occupation, industry, exposure to risk factors, and stability) in 8 economically active population aged ≥15 years in Korea. The KWCS selected households from the 2005 Population and Housing Census; individuals who met criteria for the definition of “economically active population” underwent one-on-one interviews conducted by a professional interviewer at their home. Statistics Korea accredited the reliability of KWCS in order to increase the usage of the data it collected: the survey’s response rate was 0.354, the cooperation rate was 0.662, and the refusal rate was 0.180 [19]. The final sample size was 50,032 individuals; of these, 35,902 were employed workers receiving wages. These individuals were finally selected for examination in the present study after excluding non-wage workers, such as self-employed persons without employees or self-employed persons with employees. Weighted statistical analysis was performed to prevent bias and holistically represent working conditions in Korea.

### Variables

#### Independent variables

##### General characteristics

Following the KWCS, factors relating to general employee characteristics (sex, age, income, education, alcohol, and smoking) were classified as independent variables. Each variable was defined on the basis of the survey contents. Participants were divided into the following 5 age groups: 15–29, 30–39, 40–49, 50–59, and ≥60 years. Monthly income was categorized into the following 4 groups: ≤990,000 Korean won, 1,000,000–1,990,000 won, 2,000,000–2,990,000 won, and ≥3,000,000 won. Education was categorized into the following 3 groups: middle school completion or less, high school completion, and college completion or above. Alcohol consumption was categorized as follows: none, one drink per week or less, and two or more drinks per week. Smoking status was categorized as follows: non-smoker, ex-smoker, and current-smoker.

##### Occupational characteristics

Following the KWCS, factors reflecting occupational characteristics were classified as occupational independent variables (occupation, employment type, work hours per week, tenure, shift work, and workplace scale). Variables were defined according to the KWCS contents. Occupations were categorized into the following 3 groups: white-collar workers (professional and technical occupations, higher administrator occupations, clerical occupations), services, sales workers (service occupations, sales occupations,), and blue-collar workers (skilled, semi-skilled, unskilled and agriculture, forestry, and fishery). Employment type was categorized into either regular positions or temporary or part-time positions. Work hours per week were categorized into two groups with 45 h(median) per week as the cut-off point. Tenure (number of years worked) was categorized into two groups with one year as the cut-off point. Workers were categorized as shift or non-shift workers. Those who answered ‘Yes’ to ‘I do shift work’ of the KWCS survey question ‘How is your work type?’ were classified as shift workers. This corresponds to the previously defined shift work with a narrow meaning. Workplace scale was categorized into the following 4 groups based on the number of workers employed: ≤99, 100–999, ≥1000 workers, and unknown.

Working time quality were set as major variables. Working time quality included working at night, working in the evening, working on Sundays, working on Saturdays, and working more than 10 h a day. Those who did not write 0 days in response to the KWCS question ‘When working for a minimum of 2 hours between 10 pm and 5 am is considered as working at night, how many days do you work at night?’ were considered as working at night. Those who did not write 0 day to the KWCS question ‘When working for a minimum of 2 hours between 6 pm and 10 pm is considered as working in the evening, how many days do you work in the evening?’ were considered as working in the evening. Those who did not write 0 days in response to the KWCS question ‘How many Sundays did you work in the past month?’ were considered as working on Sundays. Those who did not write 0 days in response to the KWCS question ‘How many Saturdays did you work in the past month?’ were considered as working on Saturdays. Those who did not write 0 days in response to the KWCS question ‘How many days did you work for longer than 10 hours in the past month?’ were considered as working more than 10 h a day.

#### Dependent variables

For self-perceived health, those who answered ‘Very good’ and ‘Good’ to the KWCS question ‘How is your overall health?’ were considered as having good self-perceived health.

##### Statistical analysis

To compare the variables and the characteristics of the groups with good and poor self-perceived health, univariate analysis using the Chi-square test was performed (Table 1).

**Table 1:** General characteristics and occupational characteristics of study subjects by self-perceived health

Characteristics	N	Percent	Self-perceived health		*p*-value
			Good (%)	Bad (%)	
Sex					0.142
Male	21,286	59.3	15,202(71.4)	6084(28.6)	
Female	14,616	40.7	10,334(70.7)	4282(29.3)	
Age (year)					<0.001
15-29	5588	15.6	4529(81.0)	1059(19.0)	
30-39	10,971	30.6	8370(76.3)	2601(23.7)	
40-49	10,488	29.2	7439(70.9)	3049(29.1)	
50-59	6059	16.9	3790(62.6)	2269(37.4)	
60-	2797	7.8	1408(50.3)	1389(49.7)	
Income (won)					<0.001
-990,000	4150	11.6	2610(62.9)	1540(37.1)	
1,000,000-1,990,000	13,533	37.7	9455(69.9)	4078(30.1)	
2,000,000-2,990,000	10,085	28.1	7427(73.6)	2658(26.4)	
3,000,000-	8134	22.7	6044(74.3)	2090(25.7)	
Education					<0.001
Middle school graduation or less	3894	10.8	1989(51.1)	1905(48.9)	
High graduation	13,155	36.6	9222(70.1)	3933(29.9)	
University graduation or more	18,853	52.5	14,325(76.0)	4528(24.0)	
Alcohol					0.249
No	8215	22.9	5676(69.1)	2539(30.9)	
≤ Once per week	18,231	50.8	13,232(72.6)	4999(27.4)	
≥ Twice per week	9456	26.3	6628(70.1)	2828(29.9)	
Smoking					0.661
Non-smoker	19,613	54.8	13,939(71.1)	5674(28.9)	
Ex-smoker	4037	11.2	2859(70.8)	1178(29.2)	
Current-smoker	12,252	34.1	8738(71.3)	3514(28.7)	
Occupation					<0.001
White collar	13,776	38.4	10,547(76.6)	3229(23.4)	
Service, sales worker	9846	27.4	7088(72.0)	2758(28.0)	
Blue collar	12,280	34.2	7902(64.3)	4378(35.7)	
Employment type					<0.001
Regular	28,542	79.5	21,002(73.6)	7540(26.4)	
Temporary or part-time	7360	20.5	4533(61.6)	2827(38.4)	
Work hours per week (hours)					<0.001
≤ 45 h	16,173	45	12,089(74.7)	4084(25.3)	
> 45 h	19,729	55	13,447(68.2)	6282(31.8)	
Tenure(years)					0.247
≥ 1 yr	30,672	85.4	21,781(71.0)	8891(29.0)	
< 1 yr	5230	14.6	3755(71.8)	1475(28.2)	
Shift work					0.001
No	32,533	90.6	23,225(71.4)	9308(28.6)	
Yes	3368	9.4	2310(68.6)	1058(31.4)	
Workplace scale (person)					0.342
~99	29,253	81.5	20,708(70.8)	8545(29.2)	
100~999	4132	11.5	3084(74.6)	1048(25.4)	
999~	1353	3.8	1024(75.7)	329(24.3)	
Unknown	1163	3.2	719(61.8)	444(38.2)	
Work at night					<0.001
No	31,278	87.1	22,537(72.1)	8741(27.9)	
Yes	4624	12.9	2999(64.9)	1625(35.1)	
Work in the evening					<0.001
No	19,283	53.7	14,074(73.0)	5209(27.0)	
Yes	16,619	46.3	11,462(69.0)	5157(31.0)	
Work on Sundays					<0.001
No	28,035	78.1	20,336(72.5)	7699(27.5)	
Yes	7868	21.9	5200(66.1)	2668(33.9)	
Work on Saturdays					<0.001
No	15,074	42	11,205(74.3)	3869(25.7)	
Yes	20,827	58	14,330(68.8)	6497(31.2)	
Working more than 10 h a day					<0.001
No	21,035	58.6	15,494(73.7)	5541(26.3)	
Yes	14,867	41.4	10,042(67.5)	4825(32.5)	
Total	35,902	100.0	25,536(71.1)	10,366(28.9)	

Pearson’s chi-squared test

The variables showing significance in the univariate analysis were used in the multivariate logistic regression analysis to examine the association between working time quality and self-perceived health (Table 2).

**Table 2:** Odds ratio of selected variables and self-perceived health

Variable	Adjusted	
	OR	95% CI
Sex		
Female	1.00	
Male	0.88	0.83 ~ 0.93
Age (year)		
15-29	1.00	
30-39	1.45	1.33 ~ 1.58
40-49	1.87	1.72 ~ 2.04
50-59	2.44	2.22 ~ 2.68
60-	3.12	2.77 ~ 3.51
Income (won)		
-990,000	1.00	
1,000,000-1,990,000	0.97	0.89 ~ 1.06
2,000,000-2,990,000	0.91	0.82 ~ 1.00
3,000,000-	0.88	0.79 ~ 0.98
Education		
Middle school graduation or less	1.00	
High graduation	0.69	0.63 ~ 0.75
University graduation or more	0.69	0.62 ~ 0.76
Occupation		
White collar	1.00	
Service, sales worker	0.96	0.90 ~ 1.03
Blue collar	1.11	1.04 ~ 1.20
Employment type		
Regular	1.00	
Temporary or part-time	1.35	1.27 ~ 1.44
Work hours per week (hours)		
≤ 45 h	1.00	
> 45 h	1.33	1.25 ~ 1.40
Shift work		
No	1.00	
Yes	0.73	0.67 ~ 0.81
Work at night		
No	1.00	
Yes	1.27	1.17 ~ 1.38
Work in the evening		
No	1.00	
Yes	1.15	1.08 ~ 1.23
Work on Sundays		
No	1.00	
Yes	1.13	1.06 ~ 1.20
Working more than 10 h a day		
No	1.00	
Yes	1.11	1.04 ~ 1.18

Odds ratios and 95% confidence intervals were estimated by a multiple logistic regression model including all the variables in the table

Statistical significance was set at *p* < 0.05 and SPSS v.23.0 was used for all statistical analyses.

## Results

### General characteristics and occupational characteristics of study subjects

The variables showing significance for self-perceived health in univariate analysis using the Chi-square test were age, income, education, occupation, employment type, work hours per week, and shift work. And working time quality showed a significant association with self-perceived health (working at night, working in the evening, working on Sundays, working on Saturdays, and working more than 10 h a day). Sex, alcohol, smoking, tenure, and workplace scale did not show statistical significance (Table 1).

### Relationship between working time quality and self-perceived health

Multivariate logistic regression analysis was conducted, with adjustments for age, income, education, occupation, employment type, work hours per week, and shift work, which showed statistical significance from Chi-square test. Sex was not statistically significant in chi-square test but included in covariates because it is a very important factor in self-perceived health [11–14]. After adjusting these variables for logistic regression analysis, working at night, working in the evening, working on Sundays, and working more than 10 h a day showed significant association with self-perceived health (Table 2). Working on Saturdays was eliminated in the backward stepwise selection process.

## Discussion

This study was conducted to examine the association between working time quality and self-perceived health. A novel finding of our study is that the working time quality (working at night, working in the evening, working on Sundays, and working more than 10 h a day) were significantly associated with self-perceived health.

Among previous studies on work hours, one study [18] was similar to ours, covering work hours and self-perceived health. However, our study focused on the quality of working time and not the number of work hours. When this phenomenon was viewed from the number of work hours in previous studies, working short and late hours without daytime work and working short hours on weekends after a break during weekdays cause no issue from the quantitative perspective of work hours, but is a problem from our study perspective. Although these cases have a short working hours, working at late hours and working on weekends themselves can affect ‘health at the level of quality of life’. Therefore, to accurately consider these circumstances, classifying the time of work according to working time quality will be appropriate for our study objective, as it allows a more detailed observation of this phenomenon from the perspective of ‘health’ as set out in background.

It is important to review previous findings in order to understand the association between other variables and self-perceived health. In this study, the variables showing statistical significance with self-perceived health in multivariate logistic regression analysis, after adjusting for the general characteristics, were age, occupation, employment type, work hours per week. As age increased, the odds ratio(OR) of having poor self-perceived health also increased. This is consistent with a previous study showing that good self-perceived health was associated with young age [14]. In occupation, blue collar had higher odds ratio of having poor self-perceived health than white collar, which is consistent with previous studies reporting that manual workers had poor self-assessed health compared to non-manual workers [16]. In terms of employment type, temporary workers and day-to-day workers had higher odds ratio of having poor self-perceived health than full-time workers, which is consistent with previous studies reporting that temporary/day-to-day and non-regular workers had poor self-assessed health compared to full-time workers [16]. This reflects earlier findings on the existence of inequality among workers, with non-regular workers reporting poor subjective health compared to full-time workers [20]. Work hours per week were categorized into two groups with 45 h per week as the cut-off point. Because this study focuses on the working time quality rather than the number of working hours, it is simply divided on the basis of median. With regards to weekly work hours, the group working for over 45 h had a higher odds ratio of having poor self-perceived health than the group working for less than 45 h, which is consistent with the previous reports that poor self-assessed health is associated with longer work hours [18].

Male, high income, high education level showed a protective effect on self-perceived health and this tendency is consistent with previous findings related to self-rated health [16]. But shiftwork also showed protective effect on self-perceived health, and it doesn’t in line with the finding that late work and night work affect self-rated health. To explain the reason for this result, it is important to review the concept of shift work.

There are various definitions of shift work, but it is likely that the collective elements in a collectively used definition can be divided into narrow and broad meaning [21]. The narrow meaning of shift work refers to an arrangement of work hours involving different employees or teams working continuously in a shift in order to increase the overall corporation work hours [15, 21, 22]. This meaning is comparable to the International Labor Organization (ILO) definition of shift work as “a method of organization of working time in which workers succeed one another at the workplace so that the establishment can operate longer than the hours of work of individual workers” [23]. According to the definition by the International Agency for Research on Cancer (IARC), the broad meaning of shift work refers to a different distribution of work hours that are not fixed according to the traditional weekly working pattern (06/07 am to 05/06 pm). It is the overall type of work hours that are special, variable, flexible, and nonstandard [24]. Accordingly, shift work refers to all types of work outside of usual weekly work hours and includes shift work with a narrow meaning as well as work undertaken at night or dawn, regardless of shift patterns [15, 22, 25]. Therefore, most schedules that do not fit the normal pattern, such as night shifts, fixed shifts, and rotation shifts are included.

Employees provided the status of their shift work in the survey, according to the narrow meaning of shift work. Therefore, those who work consistently only at night or in the evening were classified as non-shift workers. If night work is a major cause of the health effects of shift work, this measurement bias may have underestimated the impact of shift work [15, 21]. And because there is no information on past shift work records in this study, measurement bias can also occur [15, 21]. It is possible that the ‘healthy worker effect’ is more likely to occur in a situation where 2 group 2 shifts, which are longer in working hours and relatively inadequate than 3 shifts, are more common in Korea [15]. It should also be considered that a person who was unhealthy from the beginning could not apply for shift work. And once a person starts working, the unhealthy person is likely to leave the job [21]. For those with relatively short working periods, the unhealthy effects of shifts have not yet been expressed, so it may be possible to underestimate the results by grouping the entire shift work group without considering the working period [15]. For these reasons, we thought it would be a more appropriate model to look at qualitative aspects of working time than shift work, in examining associations with self-perceived health.

After adjusting for other variables in this study, working time quality of the employees (working at night OR 1.27, working in the evening OR 1.15, working on Sundays OR 1.13, and working more than 10 h a day OR 1.11) and self-perceived health showed a statistically significant association. Working on Saturdays (step1, OR 1.02, 95% CI 0.962~1.084) was eliminated in the backward stepwise selection process. To rule out the possibility of multicollinearity, we assessed the variance inflation factor (VIF), which is an index measuring how much the variance of an estimated regression coefficient increases because of collinearity. All the variables in our analyses showed adequate VIF values, since they were smaller than 1.9.

According to the definition of self-perceived health previously described, we need to consider the following two cases. First, working late hours, working on weekends, and working more than 10 h a day negatively affect physical health and self-awareness of physical health. Second, psychosocial effects, rather than physical problems, generate negative perception.

It is likely that the reason for which physical health actually degenerates is due to the effects of working at night. Working at night not only induces sleep disorders [26] but also increases the risk of cardiovascular disease [27], and is also associated with breast cancer [28] and colorectal cancer [29]. Because working at night causes chronic insomnia and extreme sleepiness by inducing sleep disorder [26], individuals may subjectively hold a negative view of their health. Furthermore, the biological mechanism underlying cardiovascular disease is the disturbance of the circadian rhythm (24-h cycle) resulting in changes such as the activation of the sympathetic nervous system, activation of hypothalamus-pituitary gland-adrenal cortex functions, inflammation, blood coagulation, and blood pressure increase [27]. Overtime is also associated with a variety of physical problems such as cardiovascular disease, musculoskeletal disorders, diabetes mellitus, and premature birth [30]. One may recognize an imbalance of the body and feel that he/she is in poor health.

Next, the reason why individuals may develop negative perceptions from psychosocial aspects rather than physical problems can be affected by working at night, working in the evening, working on Sundays, and working more than 10 h a day. These factors can lead to social barrier because the workers lose the opportunity to communicate with others, their ability to converse deteriorates, and they may experience serious problems in their social life, due to the conflict with personal time [31]. In addition, the more individuals work at night and on weekends, the higher the chance of experiencing depression symptoms [32]. Working at night and overtime induces job stress as well as depression [30, 33], and this can develop into a negative perception of one’s physical health. In the analysis of the association between working on weekends and psychosocial well-being using the World Health Organization (WHO) well-being index, the risk was significantly higher in the group working on weekends than in the group that did not [34]. Therefore, psychosocial aspects can cause a negative perception for self-perceived health. Furthermore, when the negative perception induces stress and depression and results in physical problems, it will aggravate physical health, which, in turn, negatively affects self-perceived health.

As self-perceived health is an index integrating physical factors and emotional factors, including satisfaction with life [11], it is likely that working at night, working in the evenings, and working on Sundays result in poor self-perceived health. In particular, working at night can have considerable physical and psychosocial effects, therefore, it is likely to result in a high odds ratio compared to working in the evening or working on Sundays.

However, working at night, working in the evenings, working on Sundays, and working more than 10 h a day cannot be banned in reality, therefore, the work must be properly allocated and the working schedule should be adjusted so that work conditions are not constant for certain individuals. Furthermore, the scope of accidents caused by related health effects that are recognized as industrial accidents should be expanded.

Our study has the following limitations. As it is a cross-sectional study based on a questionnaire, the explanatory power for cause-and-effect relationship is limited. Furthermore, the 2011 working conditions survey was used instead of a customized questionnaire for our study. The answers ‘very good’ and ‘good’ to the questions on self-perceived health were considered as having good self-perceived health and the remaining answers were considered as meaning poor self-perceived health. Therefore, the middle ground between good and poor is ambiguous. For designing a model for working time related factors associated with self-perceived health, studies need to be expanded both qualitatively and quantitatively.

## Conclusions

This study showed a statistically significant association between working time quality of employees with self-perceived health. The fact that working at late hours, working on Sundays, overtime, which lowers employee quality of life, is related to self-perceived health needs to be recognized and efforts should be made to improve these working conditions.

## Abbreviations

CI: Confidence Interval
IARC: International Agency for Research on Cancer
ILO: International Labor Organization
KOSHA: Korea Occupational Safety and Health Agency
KWCS: Korean Working Conditions Survey
OR: Odds ratio
OSHRI: Occupational Safety and Health Research Institute
VIF: Variance inflation factor
WHO: World Health Organization
